# Alterations in cochlear function during induced acute hyperinsulinemia in an animal model

**DOI:** 10.1016/S1808-8694(15)30530-9

**Published:** 2015-10-18

**Authors:** Roberto Dihl Angeli, Luiz Lavinsky, Alexandre Dolganov

**Affiliations:** 1 Master's degree in otorhinolaryngology, Universidade Federal do Rio Grande do Sul, UFRGS. Otorhinolaryngologist; 2 Associate professor, Faculdade de Medicina da Universidade Federal do Rio Grande do Sul, UFRGS. Professor of the graduate course in otorhinolaryngological surgery, Universidade Federal do Rio Grande do Sul. Otorhinolaryngologist; 3 Associate researcher of the Laboratório de Neurociências, Instituto de Pesquisas Biomédicas da Pontifícia Universidade Católica do Rio Grande do Sul, PUCRS. Neurologist

**Keywords:** audiometry, cochlea, evoked response

## Abstract

Hyperinsulinism secondary to peripheral insulin resistance has been described as the most frequent etiologic factor in cochlear and vestibular syndromes.

**Aim:**

This experimental study recorded and analyzed evoked auditory potential changes using transtympanic electrocochleography (EcochG) during induced acute hyperinsulinism in an animal model.

**Materials and Methods:**

Six adult male sheep were randomly divided into 2 groups. The animals were submitted to EcochG under general anesthesia, and a peripheral blood sample was collected to measure glycemia and insulinemia. Animals in the intervention group (n=3) received regular human insulin IV (0.1 U/kg). The control group (n=3) received saline solution. Glycemia and insulinemia were measured simultaneously with the recording of evoked potentials at 10-minute intervals during 90 minutes.

**Results:**

The intervention group experienced a progressive suppression in action potential amplitude when compared to the control group (p=0.001).

**Conclusion:**

Data strongly suggest that acute induced hyperinsulinism suppresses cochlear function. Results may be attributed to loss of Na+K+ATPase activity in the stria vascularis, leading to loss of endocochlear potential and subsequent depolarization of cochlear hair cells as well as of neural cells in the auditory portion of cranial nerve VIII.

## INTRODUCTION

Many experimental and clinical studies have demonstrated the effect of carbohydrate metabolism on inner ear function. Most of these studies have shown that hyperinsulinemia is the most frequent metabolic alteration associated with cochleo-vestibular disease.^1^, ^2^, ^3^, ^4^, ^5^, ^6^ D'Avila and Lavinsky studied the glucose profile of patients with Ménière's disease and showed that 72% of these cases had variables degrees of hyperinsulinemia that could be detected by the 5-hour glucose tolerance test.^7^

Hyperinsulinemia is an early change in peripheral resistance to insulin.^8^,^9^ Although classified as a mild disorder, it may affect significantly the inner ear metabolism and fluid concentrations.^10^

The inner ear is not able to store much energy.^11^,^12^ Paradoxically, it has a high metabolic activity, especially for maintaining endolymph ionic concentrations.^13^,^14^ Mendelsohn and Roderique showed that induction of hypoglycemia in rodents reduces significantly endolymph potassium concentrations.^15^

Mangabeira-Albernaz and Fukuda have suggested that hyperinsulinemia could block (Na+/K+)-ATPase enzyme activity on the vascular stria,^3^ removing potassium from endolymph, retaining sodium, and thus increasing the osmotic pressure at this level.^10^

Disagreements about the true impact of metabolic disorders on the cochleo-vestibular system arise from the impossibility of observing histopathological changes in vivo.^16^ The purpose of this study was monitor the changes in evoked potentials by means of transtympanic electrocochleography (ECoG) during acute induction of hyperinsulinemia in sheep with the aim of understanding this association in greater depth.

## MATERIAL AND METHOD

This experimental study was carried out in the facilities of the animal experimental unit of the research center of the institution. The Research Ethics Committee approved the study (registration number 04-070).

The sample size was based on the estimated difference of at least 3.5 standard deviation units between groups; the alpha value was set at 0.05 and the statistical power of the study was defined as 80%.

Six adult male Corriedale sheep weighing from 40 to 43 kg were randomly allocated to two equal groups (control and intervention groups). International guidelines for handling experimental animals were followed throughout the study.

### Anesthesia

Animals required a 48-hour fasting period prior to anesthesia due to slow gastric emptying in ruminants.

**Figure 1:**
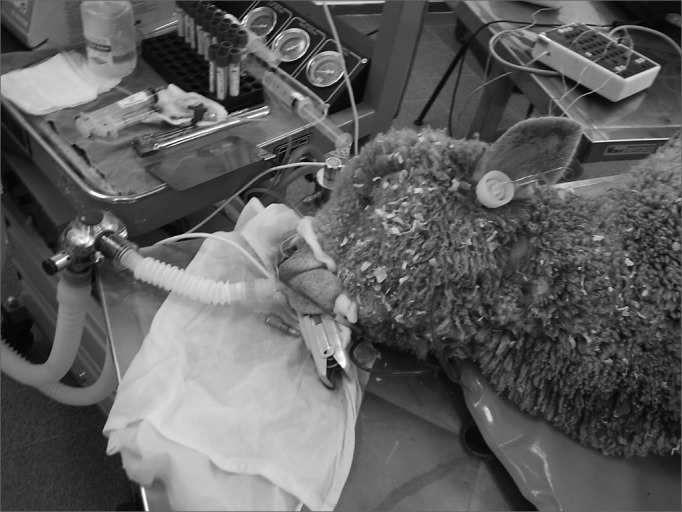
Study animal during the experimentation phase.

Sedation was attained with 500 mg/kg acepromazine (Univet, Sao Paulo, SP). Induction of anesthesia was done with 15 mg/kg intravenous sodium thiopental (Abbott, Rio de Janeiro, RJ) and maintained with the same drug by a continuous infusion pump (Nutrimat II, B. Braun, Sao Gonçalo, RJ) at 600 mg/hour, supervised by a veterinary doctor. Orotracheal intubation was done in all animals with an 8 or 9 mm tube (Fig. 1) coupled to a closed system anesthesia cart (Modulus 4000, Narcosul, Porto Alegre, RS) with 100% oxygen. Oxygen saturation, the heart rate (Biox 3700e, Ohmeda, Louisville, CO, EUA), the tidal volume, the respiratory frequency (Ventcare, São Paulo, SP), and the anal temperature were monitored.

### Recording evoked potentials

**Figure 2:**
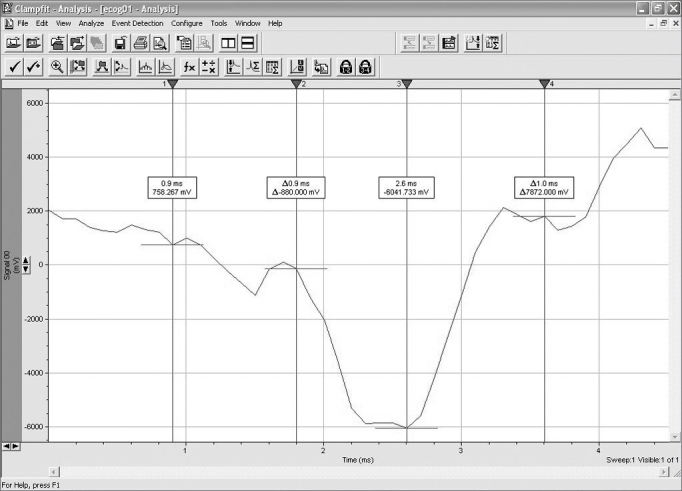
Interface of the Clampfit 9.2 software.

Auditory evoked potentials were recorded using a Morpheu All-In-One system developed by Dolsch Biomedical (Porto Alegre, RS). These recordings were analyzed using the Clampfit 9.2 software developed by Axon Instruments (Fig. 2).

The primary puncture electrode (Medelec Ltd) was inserted into the middle ear through a small tympanotomy; its tip touched the promontory. The secondary electrode was placed on the nasal mucosa.

A microphone located on the external acoustic meatus issued a positive polarity (condensation) 110 dBHL acoustic stimulus. Tested frequencies were 5, 6, 7, 8, 9 and 10 kHz. Fifteen and 15.000 Hz filters were used. The acoustic signal was amplified 100,000 times; 500 stimuli were provided for each frequency, totaling 3,000 stimuli for each one of the 10 recording moments.

### Glucose and insulin blood samples

Peripheral blood was taken by jugular puncture to measure blood glucose and insulinemia. Blood glucose was measured using the enzymatic-colorimetric glucose-oxidase (Labtest) method; insulinemia was measured with electrochemoluminescence immunoassays using Modular Analytics E170 (Roche) analyzers. All laboratory tests were carried out at the Clinical Pathology Laboratory of the institution.

### Induction of acute hyperinsulinemia

Blood samples were taken before the intervention to measure blood glucose and insulinemia simultaneously with evoked potential recordings.

Study group animals (n=3) were given 0.1 U/kg intravenous regular insulin in 20 ml of saline. Control group animals (n=3) were given only 20 ml of saline. Evoked cochlear potentials were recorded every 10 minutes during 1 hour and 30 minutes (90 minutes), totaling 10 data gathering moments. At the same time, peripheral blood was taken to measure blood glucose and insulinemia curves.

### Statistical analysis

The Statistical Package for the Social Sciences (SPSS) version 12.0 software was used for data analysis. Baseline data (gathered at time zero) of the two groups were compared applying the Student t test for independent samples. Analysis of Variance (ANOVA) for repeated samples was applied to assess amplitude changes in the cochlear action potential (AP) and blood glucose levels. Recordings of the cochlear action potential (AP) were presented as folds, the ratio of values observed at different moments from baseline measurements. The software SigmaPlot version 8.0 was used to prepare the graphic presentation of results.

The significance level was defined as p < 0.05.

## RESULTS

There were no losses during the experimentation phase. Animals were extremely docile throughout the experimentation phases, which facilitated anesthetic and surgical instrumentation.

Recovery from anesthesia in the research center took on average 2 hours, after which the animals were transported to their origin under the care of the supplier.

**Figure 3:**
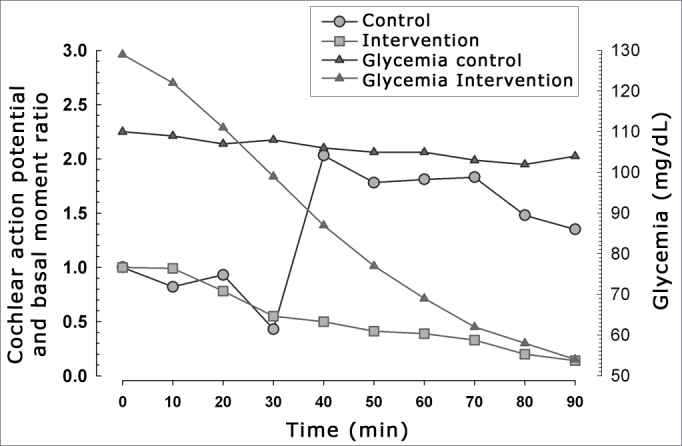
Relation between cochlear action potential amplitude and blood glucose levels in both groups.

**Figure 4:**
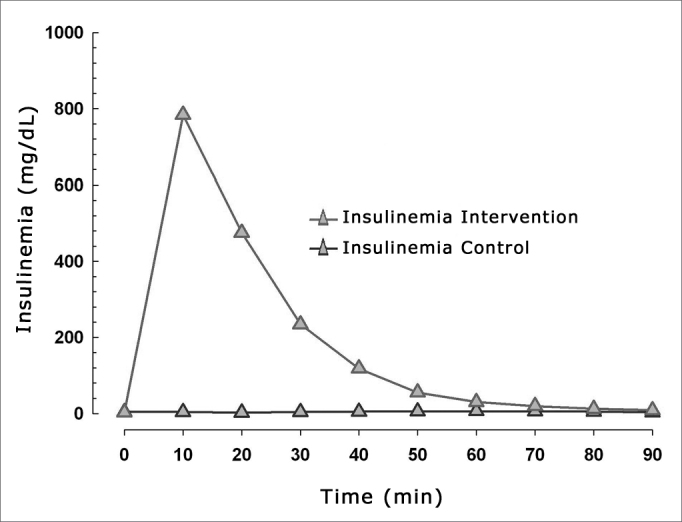
Mean blood insulin levels in the control and intervention groups.

Hyperinsulinemia and hypoglycemia were effectively induced (Figure 3, Figure 4). Figure 3 shows the relation between mean blood glucose levels during the experiment and the mean amplitude of the cochlear AP in both groups.

Baseline data for both groups were similar in terms of insulinemia, blood glucose, and amplitude of the cochlear AP (respective p values – 0.59, 0.14 and 0.11).

**Figure 5:**
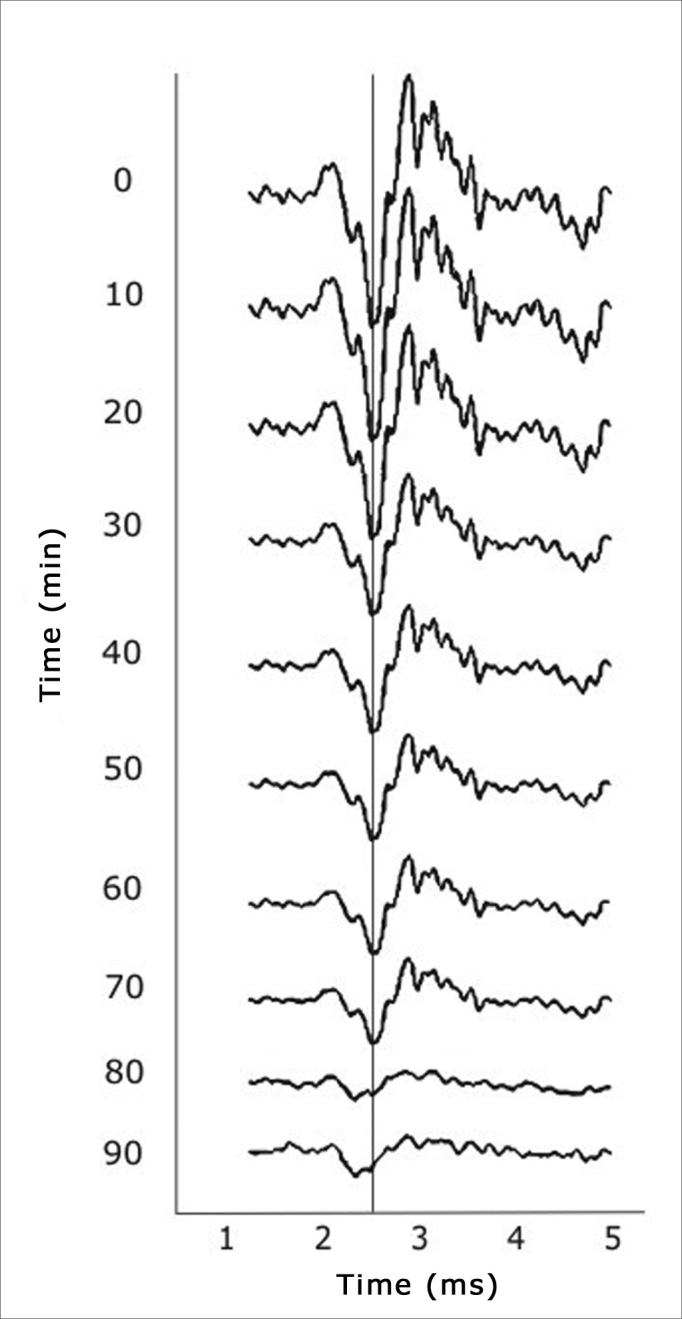
Tracing of animals in the intervention group showing progressive reductions in the amplitude of cochlear action potentials.

The ANOVA test for repeated measures of AP amplitudes revealed that the intervention groups had a significant decrease in the amplitude of the cochlear PA compared to the control group (p=0.001). Figure 5 shows the tracings belonging to one of the intervention animals, showing the progression of the amplitude across 90 minutes.

## DISCUSSION

The research group has used sheep as experimental animals for otological surgical research and training since the last decade.^17^, ^18^, ^19^, ^20^ Anatomical and morphometric similarities between sheep and human ears have been demonstrated.^21^ Structures are comparable in size to those of humans, which made it possible to use conventional anesthetic and surgical tools, without any adaptation.

Most of the success in handling these animals was the results of professional support by qualified and well-trained veterinary doctors, based on their experience with previous protocols developed by the research group.

In this study, electrocochleography was applied to monitor the response to acute hyperinsulinemia. The literature contains no references about this method in sheep, making the present study a pioneering investigation in the context of this theme. The experience gained by the authors will enable other projects to be developed to assess other components of auditory evoked potentials, such as cochlear microphonism and the sum potential. A true mapping or scan was undertaken as the frequency spectrum at which stimulation would provide consistent responses was unknown; the frequencies chosen were those from 5 to 10 kHz. Thus, tone bursts, rather than broadband clicks (BBC), were chosen as the stimulus.

The idea that hyperinsulinemia is the first step in the development of peripheral insulin resistance (or glucose intolerance) has opened a vast field off research about its clinical repercussions. The negative impact of hyperinsulinemia on inner ear homeostasis is probably due to the ionic and metabolic features of the vascular stria, which is responsible for maintaining the endocochlear potential by secreting potassium into the endolymphatic space.

Our results revealed a much wider variation in blood insulin values compared to variation in blood glucose levels (Fig. 4), suggesting that the effects of exposure are secondary to hyperinsulinemia, and to a lesser degree, to hypoglycemia.

The predominant electrical response was a progressive decrease in the amplitude of the neural AP in the intervention group. Two mechanisms may explain this phenomenon:

• Hyperinsulinemia acts directly on auditory nerve fibers, compromising the ionic transport across the cell membrane and interrupting depolarization.
• Hyperinsulinemia acts on the vascular stria, compromising the maintenance of the endocochlear potential, thus interruption hair cell depolarization; this in turn affects subsequent depolarization of neural fiber cells.

We strongly believe that the neural effect we observed only reflects interference on cochlear activity secondary to induced acute hyperinsulinemia. Recent studies by our group have shown that the cochlear activity in sheep, measured by distortion product otoacoustic emissions (DPOAE), decreased significantly after induction of acute hyperinsulinemia,^17^,^18^ suggesting a cochlear, rather than a neural, effect.

The theoretical mode that best explains our results is blockage of the (Na+/K+)-ATPase enzyme on the vascular stria, as suggested by Mangabeira-Albernaz and Fukuda.^3^ This effect alters the ionic composition of endolymph, interrupting full depolarization of inner hair cells and of fibers comprising the auditory portion of the VIII cranial nerve.

Lack of synchronism between the period of maximum hyperinsulinemia and the period of lowest AP amplitude may be explained theoretically by the fact that enzyme blockage has a somewhat delayed effect on endolymphatic potassium concentrations; enough molecules remain to depolarize the cells in the first few minutes.

Insulin peripheral resistance and hyperinsulinemia have been studied in detail in a cardiovascular context, but are rarely associated with inner ear disorders. Studies undertaken by this and other groups^3^,^17^,^18^,^22^,^23^ have shown that inner ear disease may be interpreted as an early warning sign for insulin resistance, justifying further clinical investigation in daily medical practice.

## CONCLUSION

Our results demonstrated that induction of acute hyperinsulinemia results in marked changes in auditory function, evidenced by a decreased amplitude of the cochlear AP. These results may be attributed to suppression of (Na+/K+)-ATPase enzyme activity on the vascular stria, resulting in loss of the endocochlear potential and interruption of depolarization of fibers comprising the auditory portion of the VIII cranial nerve.

## References

[1] Updegraff WR (1977). Impaired carbohydrate metabolism and idiopathic Meniere's disease. Ear Nose Throat J..

[2] Proctor CA (1981). Abnormal insulin levels and vertigo. Laryngoscope..

[3] Mangabeira Albernaz PL, Fukuda Y (1984). Glucose, insulin and inner ear pathology. Acta Otolaryngol..

[4] Kirtane MV, Medikeri SB, Rao P (1984). Blood levels of glucose and insulin in Meniere's disease. Acta Otolaryngol. Suppl.

[5] Kazmierczak H, Doroszewska G (2001). Metabolic disorders in vertigo, tinnitus, and hearing loss. Int Tinnitus J..

[6] Lavinsky L, Oliveira MW, Bassanesi HJ, D'Avila C, Lavinsky M (2004). Hyperinsulinemia and tinnitus: a historical cohort. Int Tinnitus J..

[7] D'Avila C, Lavinsky L (2005). Glucose and insulin profiles and their correlations in Méniére's disease. Int Tinnitus J..

[8] Kraft JR (1975). Detection of diabetes mellitus in situ (occult diabetes). Lab Med..

[9] (1979). Classification and diagnosis of diabetes mellitus and other categories of glucose intolerance. National Diabetes Data Group. Diabetes..

[10] D'Ávila C, Lavinsky L, Lavinsky L (2006). Tratamento em otologia.

[11] Morizane I, Hakuba N, Shimizu Y, Shinomori Y, Fujita K, Yoshida T (2005). Transient cochlear ischemia and its effects on the stria vascularis. Neuroreport..

[12] Yamamoto H, Makimoto K (2000). Sensitivity of the endocochlear potential level to cochlear blood flow during hypoventilation. Ann Otol Rhinol Laryngol..

[13] Kuijpers W, Bonting SL (1696). Studies on (Na+-K+)-activated ATPase. XXIV. Localization and properties of ATPase in the inner ear of the guinea pig. Biochim Biophys Acta..

[14] Wangemann P (2006). Supporting sensory transduction: cochlear fluid homeostasis and the endocochlear potential. J Physiol..

[15] Mendelsohn M, Roderique J (1972). Cationic changes in endolymph during hypoglycemia. Laryngoscope..

[16] Bittar RSM, Bottino MA, Simoceli L, Venosa AR (2004). Labirintopatia secundária aos distúrbios do metabolismo do açúcar: realidade ou fantasia?. Rev Bras Otorrinolaringol..

[17] Zuma e Maia FC, Lavinsky L (2006). Distortion product otoacoustic emissions in an animal model of induced hyperinsulinemia. Int Tinnitus J..

[18] Maia FCZ, Lavinsky L, Mollerke RO, Duarte MES, Pereira DP, Maia JE (2008). Distortion product otoacoustic emissions in sheep before and after hyperinsulinemia induction. Braz J Otorhinolaryngol..

[19] Lavinsky L, Goycoolea M, Tos M, Thompson J (1997). Otitis media today.

[20] Lavinsky L, Goycoolea M, Ganança MM, Zwetsch Y (1999). Surgical treatment of vertigo by utriculostomy: an experimental study in sheep. Acta Otolaryngol..

[21] Seibel VA, Lavinsky L, de Oliveira JA (2006). Morphometric study of the external and middle ear anatomy in sheep: a possible model for ear experiments. Clin Anat..

[22] Lavinsky M, Wolff FH, Lavinsky L (2000). Estudo de 100 pacientes com clínica sugestiva de hipoglicemia e manifestações de vertigem, surdez e zumbido. Rev Bras Med Otorrinolaringol..

[23] Lehrer JF, Poole DC, Seaman M, Restivo D, Hartmann K (1986). Identification and treatment of metabolic abnormalities in patients with vertigo. Arch Intern Med..

